# Genetic Diversity of Seven Cattle Breeds Inferred Using Copy Number Variations

**DOI:** 10.3389/fgene.2018.00163

**Published:** 2018-05-15

**Authors:** Magretha D. Pierce, Kennedy Dzama, Farai C. Muchadeyi

**Affiliations:** ^1^Animal Production, Agricultural Research Council, Pretoria, South Africa; ^2^Department of Animal Sciences, University of Stellenbosch, Stellenbosch, South Africa; ^3^Biotechnology Platform, Agricultural Research Council, Pretoria, South Africa

**Keywords:** genetic diversity, CNVs, population structure, South African cattle, breed history, selection

## Abstract

Copy number variations (CNVs) comprise deletions, duplications, and insertions found within the genome larger than 50 bp in size. CNVs are thought to be primary role-players in breed formation and adaptation. South Africa boasts a diverse ecology with harsh environmental conditions and a broad spectrum of parasites and diseases that pose challenges to livestock production. This has led to the development of composite cattle breeds which combine the hardiness of Sanga breeds and the production potential of the Taurine breeds. The prevalence of CNVs within these respective breeds of cattle and the prevalence of CNV regions (CNVRs) in their diversity, adaptation and production is however not understood. This study therefore aimed to ascertain the prevalence, diversity, and correlations of CNVRs within cattle breeds used in South Africa. Illumina Bovine SNP50 data and *PennCNV* were utilized to identify CNVRs within the genome of 287 animals from seven cattle breeds representing Sanga, Taurine, Composite, and cross breeds. Three hundred and fifty six CNVRs of between 36 kb to 4.1 Mb in size were identified. The null hypothesis that one CNVR loci is independent of another was tested using the *GENEPOP* software. One hunded and two and seven of the CNVRs in the Taurine and Sanga/Composite cattle breeds demonstrated a significant (*p* ≤ 0.05) association. *PANTHER* overrepresentation analyses of correlated CNVRs demonstrated significant enrichment of a number of biological processes, molecular functions, cellular components, and protein classes. CNVR genetic variation between and within breed group was measured using phiPT which allows intra-individual variation to be suppressed and hence proved suitable for measuring binary CNVR presence/absence data. Estimate PhiPT within and between breed variance was 2.722 and 0.518 respectively. Pairwise population PhiPT values corresponded with breed type, with Taurine Holstein and Angus breeds demonstrating no between breed CNVR variation. Phylogenetic trees were drawn. CNVRs primarily clustered animals of the same breed type together. This study successfully identified, characterized, and analyzed 356 CNVRs within seven cattle breeds. CNVR correlations were evident, with many more correlations being present among the exotic Taurine breeds. CNVR genetic diversity of Sanga, Taurine and Composite breeds was ascertained with breed types exposed to similar selection pressures demonstrating analogous incidences of CNVRs.

## Introduction

Copy number variations are deletions, duplications, and insertions larger than 50 bp in size that modify the DNA structure and play a significant role in the genomic variability and hence diversity evident within and among breeds (Letaief et al., 2017). They have been observed to affect a greater percentage of genomic sequences relative to other forms of genomic variations like single nucleotide polymorphisms (SNPs) (Zhang et al., 2009; Hou et al., 2012; Liu and Bickhart, 2012). SNPs and microsatellite analyses have been used to assess population structures and genetic diversity in order to gain insight into origin, history and adaptation of cattle. CNVR loci have however been found within gene boundaries, with the incidence of some coinciding with breed histories and breed formation patterns (Matukumalli et al., 2009; Hou et al., 2011). Covering a greater number of sequences than SNPs, CNVs may alter gene dosage, disturb coding sequences or sway gene regulation (Stranger et al., 2007). CNVs have been proposed to play a role in genetic adaptation (Liu et al., 2010). Stranger et al. (2007) demonstrated SNPs and CNVs to capture 83.6 and 17.7% of the observed genetic variation respectively with very little overlap in the variation captured by the two variant types. It was thus hypothesized that ascertaining the genetic variations captured by CNVs will generate supplementary information regarding the genetic variation which may add to that already obtained from SNPs. CNVs may hence be a suitable genomic marker for ascertaining cattle origins and history as well as divergence amongst breeds.

The formation and fixation of CNVRs within the genome has not been fully explored. It has been proposed that forces such as recombination, selection and mutations are the primary factors driving the genomic architecture of large variations (Jimenez, 2014). Their fixation within the genome indicates an advantage that necessitates DNA repair mechanisms to not remove them from the genome. Gene ontology analyses demonstrate CNVRs to be prevalent in specific regions of the genome covering genes involved in specific biological, cellular or molecular process (Wang et al., 2015). Whether the fixation of CNVRs at one region of the genome corresponds with the fixation of another CNVR at a different region but possibly involved in the same process or a confounding process has not been explored. If CNVRs are correlated within the genome, this may indicate them to not be random events that occur subsequent to recombination errors, but that selection pressure and other biological mechanisms may be driving their formation and/or fixation at specific locations within the genome.

A number of Taurine, Sanga, and Composite breeds are found in South Africa. While exotic Taurine breeds demonstrate improved production subsequent to the development and elevated focus of intense selection programs, indigenous Sanga breeds of South Africa are recognized for their innate ability to handle the range of harsh climatic conditions, feed, and water scarcity together with a widespread array of diseases and pathogens customary to South Africa (Hoffmann, 2010; Mirkena et al., 2010). Composite breeds, like the Bonsmara have been developed to merge the adaptative ability of indigenous cattle with the productive ability of the Taurine breeds (Bonsma, 1980). Makina et al. (2014) assessed the genetic variation of Composite, Sanga, and Taurine cattle breeds, using genome wide SNP data. Considering the evidenced adaptation of Sanga breeds that have also been introgressed into Composite breeds, the determination of genetic variation of CNVRs in these breeds may hold further insight into understanding the multiple components of functional breed diversity and the subsequent implications thereof. This may have important inference on current breed management and genetic improvement practices. In addition to this, ascertaining whether or not the presence of one CNVR within the genome is correlated with another CNVR would give further insight into understanding the driving force behind CNVR formation and possible fixation within the genome.

This study therefore comprised an investigation into the diversity of seven cattle breeds sampled in South Africa (Angus, Drakensberger, Afrikaner, Holstein, Nguni, and Bonsmara) from each of three breed groups (Taurine, Sanga, and Composite) and one cross breed (Nguni X Angus) utilizing CNVRs. It was hypothesized that CNVR genetic diversity would parallel breed history and adaptation, with greater CNVR variation being present between breeds that are more distantly related or exposed to distinct selection pressures. The relationship between identified CNVRs within the genome was also explored in order to determine whether selection pressures were causing joint fixation of multiple CNVRs involved in the similar or complementary processes. Illumina BovineSNP50 genotyping methodology was used in conjunction with *PennCNV* to identify CNVRs and subsequent genes enriched by CNVRs. CNVRs were used to ascertain levels of genetic diversity and to determine the measure of pairwise correlation in CNVR presence within and among breeds.

## Materials and methods

### Sample collection and genotyping

Genomic data was obtained from Makina et al. (2014) and Makina et al. (2015). This comprised 287 animals comprising of two Taurine (45 Holstein and 32 Angus), two Sanga (59 Nguni and 48 Afrikaner), two Composite (46 Bonsmara and 48 Drakensberger) and one crossbred (10 Nguni Angus) breeds sampled from throughout South Africa. Informed consent from respective breeders was obtained. The protocol utilized for the collection of samples, DNA extraction and genotyping has been published (Makina et al., 2014, 2015). Animal handling and sample collection were performed according to the University of Pretoria Animal Ethics Committee code of conduct (E087-12).

### SNP quality control

SNP quality control was performed for all animals using *PLINK* v.1.07. Those SNPs with a MAF of <0.02, call rate of <95% and missing genotype frequency of more than 10% were excluded from further analyses. Of the 54,609 markers on the Illumina Bovine SNP50 beadchip v2, 45,924 SNPs had a call rate and MAF of greater than 0.95 and 0.02 respectively and thus remained for further analyses. Forty five thousand nine hundred and twenty-five SNPs thus remained for further analyses. A *PennCNV* input file containing LogR ratio and B allele frequency data of 45 925 good quality SNPs for 287 animals was generated in *GenomeStudio* Software 2011.1 and exported for further analyse.

### CNVRs identification and distribution

*PennCNV* has outperformed a number of CNV detection packages especially with regard to specificity and sensitivity of CNV calling (Castellani et al., 2014; Zhang Q. et al., 2014). This software was therefore utilized to identify CNVs within the genome of 287 cattle. The PennCNV compile_pfb script (Wang et al., 2015) was utilized to create a pfb file from the data. The detect_cnv.pl was run to detect CNVs on 29 autosomes. GC content within 1 Mb region (500 K per side) surrounding each marker was calculated and utilized to create the bovine gcmodel. A second analyses including the gcmodel option was also run for comparative purposes. In order to reduce the number of false positive CNVs, identified CNVs were filtered according four different filtering stringencies as described by Wang et al. (2015). All CNVs filtered in the absence of the gcmodel with a genomic waviness of 0.04 were identified by other models and were therefore used for further analyses. In addition, CNVR identified were checked for false positive CNVR reported by Zhou et al. (2016).

The bioinformatics and evolutionary genomics *VENN* diagram webtool (http://bioinformatics.psb.ugent.be/webtools/Venn/) was used to create a venn diagram demonstrating the overlap between CNVs identified in different breeds. Adjacent and overlapping CNVs were aggregated to form CNVRs utilitizing bioinformatic approaches as recommended by Redon et al. (2006).

A CNVR dataset was created from CNVRs identified in 287 animals from seven cattle breeds. CNVR were each treated as individual loci and only those CNVRs identified in three or more animals were utilized so as to reduce the rate of false positives within the dataset (Jakobsson et al., 2008). Three input files were generated. The first contained individual animals with binomial presence/absence data for each of the 110 CNVR loci that remained post pruning. The second dataset comprised of presence/absence data of the 110 CNVR loci for each of the seven cattle breeds, while the third dataset contained information on the CNVR loci frequencies for each of the seven cattle breeds.

### CNVR correlations and representation

A pairwise association testing the null hypothesis that genotypes at one locus were independent of genotypes at the other locus was performed using *GENEPOP* (Raymond and Rousset, 1995). Only those CNVR identified in three or more animals were used. CNVR correlations across all seven breeds and across Sanga/Composite and Taurine breeds were run respectively. Contingency tables, demonstrating the relationship between all pairs of loci within and between breeds was created. A markov chain algorithm described by Raymond et al. (Raymond and Rousset, 1995) computed a G-test and probability test for each table. CNVRs demonstrating a significant correlation with a *p*-value of <0.05 were uploaded onto *UCSC* to ascertain genomic region information. A *PANTHER* overpresentation analyses using the Bonferoni correction for multiple testing was performed on genes covered by correlated CNVRs to ascertain whether any molecular functions, biological processes or cellular components were significantly (*p* < 0.05) overrepresented by correlated CNVRs.

### CNVR genetic diversity analyses

Molecular variance (AMOVA) and principle component analyses were subseqeuntly performed on the pruned data comprising of 110 CNVR loci in 287 samples using *GenAlex* software (Peakall and Smouse, 2012). A tri-matrix of squared euclidean distances was used to calculate the pairwise population values (PhiPT) by means of an AMOVA using 9,999 permutations. PhiPT values, which are analogous to Wrights' F_ST_ indices, measure population genetic differentiation from binary data and were used to measure the genetic variation of CNVRs within and among cattle breeds. This measure allows intra-individual variation to be suppressed and hence proved suitable for measuring binary CNVR presence/absence data (Teixeira et al., 2014). A genetic distance trimatrix was utilized to determine standaradised eigenvectors for principle components 1–100. Eigenvalues present the amount of genetic variation contained by each respective principle component (PC). In order to determine how many PCs to contain within the model, each eigenvalue was divided by the total sum of eigenvalues in order to establish the fraction of total variance retained versus the number of eigenvalues. Kaiser's stopping rule states that only PCs demonstrating eigenvalues over 1.00 should be considered in the analysis. This comprises the most utilized method for determining the number of PCs to retain in the analyses (Peres-Neto et al., 2005).

*STRUCTURE* v2.3.4 was utilized to perform a model based clustering analyses of population structure as reported by Pritchard et al. (2000) and Falush et al. (2007). Analyses were run using a burn-in period of 5000 Reps. The model used did not assume any specific mutation process. Considering the exact mutation and inheritance patterns of CNVs is not as yet fully understood (Zhang Q. et al., 2014), it was thus deemed suitable for CNV analyses. Multiple analyses were performed for *K* = 2 to *K* = 8. The membership coefficient Q estimate matrix was plotted as a barplot.

The *R* package *hclust* was used to compute a distance matrix from binomial CNVR present/absence data for each animal which was then used to perform a hierarchal dissimilarity cluster analysis on regions with variable copy numbers. This was performed for each of the three datasets and plotted to demonstrate clusters.

### CNVR gene ontology and representation

Genomic regions of CNVRs identified were uploaded into *UCSC* and details of the regions together with the reflink and refGene genes covered were obtained. *VENN* (http://bioinformatics.psb.ugent.be/webtools/Venn/) was utilized to construct a venn diagram demonstrating the overlap of those genes enriched within CNVs identified across breeds. Gene ontologies were determined by means of the *PANTHER* databases (Helleday, 2003). The hypothesis that genes were over or under represented in *PANTHER* pathways, biological processes, cellular components, and molecular pathways was tested using the bonferoni correction at a significance level of 0.05.

## Results

### CNVRs identification and characterisation

One thousand and fifty five unique CNVs were identified in 197 of the 287 cattle. CNVs ranged from 31 kb to 2.9 Mb in size, with an average length of 301 kb (Table 1). The majority (625) of the CNVs were single copy deletions. Four hundred and five single copy duplications together with 5 double copy duplication and 20 double copy deletions were reported. The smallest CNV was a single copy duplication, while the largest was a single copy deletion.

**Table 1:** CNV summary statistics of Copy number (CN), Number of CNVs (CNVs) and maximum (MaxL), minimum (MinL) and average (AL) CNV lengths.

**CN**	**CNVs**	**MinL (bp)**	**MaxL (bp)**	**AL (bp)**
0	20	44 415	227 892	109 759.2
1	625	36 419	2 933 073	361 997.179
3	405	31 397	1 297 541	217 608.642
4	5	93 420	572 953	218 348.800
Total	1055	31 397	2 933 073	301 105.844

Adjacent and overlapping CNVs were joined to form 356 CNVRs (Additional File 1). CNVRs ranged from 36 kb to 4.1 Mb in length with an average length of 287 kb across breeds. The most CNVRs were identified on chromosomes 4 and 6, while chromosomes 22 and 28 had the least CNVRs. Chromosome 25 presented the greatest portion of its length to be covered by CNVRs. The largest CNVR was present on chromosome 11, while the smallest occurred on chromosome 1. The percentage of chromosomes covered by variations in copy number ranged from 1.15% of chromosome 28 to 14.14% of chromosome 25.

The most CNVRs were identified in the Nguni Angus breed (*n* = 114), followed by the Holstein (*n* = 102) and Angus (*n* = 101) breeds. The Nguni Angus breed also demonstrated the highest average CNVRs per animal at 11.41, considerably higher than the 1.30–3.15 averages of the remaining breeds. Despite the Nguni Angus cross having noticeably fewer animals in the study, the most CNVRs (114) were identified in these 10 animals. 102 and 101 CNVRs were identified in 45 and 32 Holstein and Angus animals respectively. The least CNVRs were identified in the 46 and 48 Bonsmara and Drakensberger animals (Table 2). The Nguni demonstrated the most CNVRs of the indigenous breeds, with an average of 1.61 CNVRs per animal.

**Table 2:** CNVR summary statistics for each of seven cattle breeds (Afrikaner–ANG, Angus–ANG, Bonsmara–BON, Drakensberger–DRK, Holstein–HOL, Nguni–NGU, and Nguni Angus cross–NGxAN).

**BRD**	**ANML**	**AN CNV**	**CNVR**	**Av**	**MinL (bp)**	**MaxL (bp)**	**AL(bp)**	**GEN**
AFR	48	31	76	1.58	36 419	4 181 753	498 498.79	96
ANG	32	25	101	3.15	42 946	4 181 753	581 476.86	430
BON	46	35	60	1.30	52 472	4 181 753	668 772.47	96
DRK	48	24	63	1.31	38 235	4 181 753	353 594.71	29
HOL	45	28	102	2.26	42 164	4 181 753	558 378.40	207
NGU	59	47	95	1.61	44 415	4 181 753	467 388.03	142
NGxAN	10	7	114	11.4	54 147	4 181 753	584 980.73	616
	287	197	356	1.29	36 419	4 181 753	535 289.93	809

**Figure 1:**
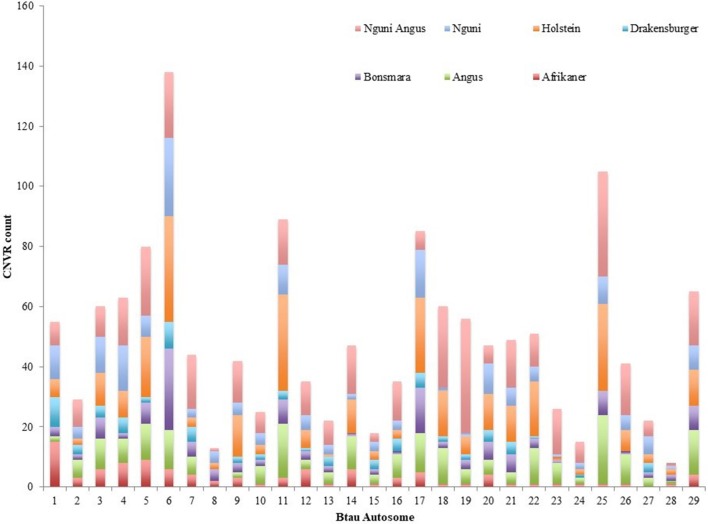
Chromosomal distribution of CNVRs for each of seven cattle breeds.

The chromosomal distribution of CNVRs across breeds demonstrates great variation in the size and number of CNVRs identified per autosome (Figure 1). Chromosomes 4 and 6 possessed the most Falush et al. (2007) CNVRs. The largest CNVR found on chromosome 11 (CNVR11) was 4.1 Mb in length. This CNVR was present in 76 animals from all 7 breeds. The smallest CNVR of 36 kb was identified in the Afrikaner cattle breed while the Bonsmara, despite demonstrating the least CNVRs, had the longest average CNVR.

**Figure 2:**
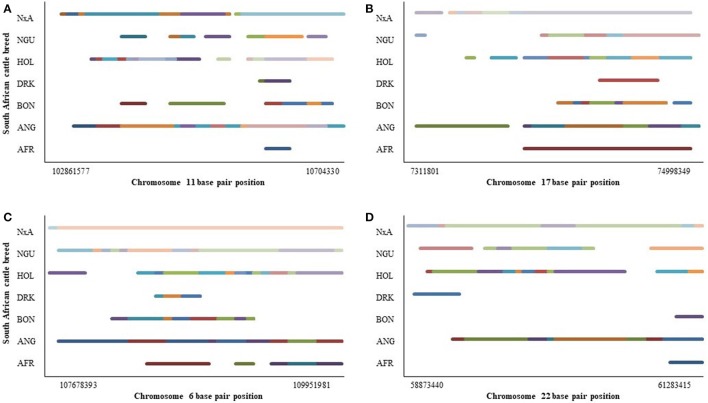
CNV chromosomal distribution in seven cattle breeds at four different chromosomal locations namely **(A)** chr11:102861577-10704330, **(B)** chr17:7311801-74998349, **(C)** chr6:107678393-109951981, and **(D)** chr22:58873440-61283415.

Only 4 CNVRs were identified in all seven cattle breeds with chromosome 17 and chromosome 11 presenting the 2 most common CNVR. Figure 2 demonstrates the spatial distribution of CNVs within each breed for the 4 mutual CNVRs that were identified in 53–78 animals. In all four instances Angus, Holstein, and Nguni X Angus CNVs represented the largest portion of the CNVR while Drakensberger CNVs denoted the least. The consequence of such discrepancies in specific CNV regionality between breeds should be investigated. Most CNVs were shared between fewer breeds with Angus and Nguni Angus breeds demonstrating the most common CNVs (Additional File 2).

### CNVR correlations

Of the 110 CNVR evident in more than 2 individuals, 22 loci demonstrated a significant pairwise association (*p* ≤ 0.05) with at least one other loci across all 7 breeds, 11 of which demonstrated highly significant correlations (*p* ≤ 0.002). These loci culminated to form 74 significant correlations with a *p*-value of < 0.05 (Additional File 3). Zhang Q. et al. (2014) report a significant reduction in the CNVR associations with increase in CNVR prevalence. Associated CNVRs in this study, however were present in 3 to 78 animals (Additional File 4). On analyzing the data independantly for each of the indigenous (Nguni, Sanga, Bonsmara, Afrikaner, Drakensberger) and exotic (Holstein, Angus) breeds, only 7 loci were significantly correlated within indigenous breeds representing 6 significant correlations, while 102 loci within the exotic Taurine breeds presented 904 significant (*p* ≤ 0.05) correlations (Additional File 5). Deletions and duplications at the same loci were treated as independent CNVRs. Only one of the correlated loci pairs of all breeds demonstrated a deletion corresponding with duplication. The rest exhibited correlations occurring between CNVRs of the same copy number. Within the 6 CNVR correlations of the indigenous Sanga and Composite breeds, 4 were between CNVR duplications and 2 were between a deletion and duplication (Additional File 6). The significant Taurine breed CNVR associations exhibited 866 deletion associations, 38 duplication associations and 2 deletion and duplication associations. The 906 correlations evident among CNVRs of Taurine breeds encompass 849 genes. The 7 CNVR correlations evident among the indigenous animals, on the other hand covered 76 genes. Genes represented within correlated CNVRs were involved in a number of biological, molecular and cellular pathways and are presented in Table 3. The representation of CNVR genes involved in processes, pathways and components that are involved in adaptation have implicated CNVRs to play a role in adaptation. The significant overrepresentaion of such ontologies represented in Table 3 by correlated CNVRs further supports this proposal.

**Table 3:** Ontologies (GO) with significant (*p* < 0.05) enrichment by genes covered by correlated CNVRs in seven South African cattle breeds.

GO^*^	**REF**	**GEN**	**EXP**	**TP**	**FOLD**	***P*-VAL**
**CC**						
Troponin complex	8	4	0.13	+	>5	9.80E-03
Intracellular organelle part	5 633	133	89.57	+	1.48	1.21E-04
Organelle part	5 796	134	92.17	+	1.45	3.80E-04
Membrane-bounded organelle	9 165	190	145.74	+	1.30	4.06E-04
Cytoplasm	7 752	160	123.27	+	1.30	1.85E-02
Intracellular membrane-bounded organelle	8 047	166	127.96	+	1.30	1.04E-02
Organelle	10 107	205	160.72	+	1.28	3.47E-04
Intracellular organelle	9 084	183	144.45	+	1.27	9.11E-03
Intracellular part	10 523	209	167.33	+	1.25	1.39E-03
Intracellular	11 092	211	176.38	+	1.20	4.64E-02
**PC**						
Translation elongation factor	50	6	0.80	+	>5	3.49E-02
**BP**						
Cellular biosynthetic process	2 369	69	37.67	+	1.83	3.09E-03
Organic substance biosynthetic process	2 450	70	38.96	+	1.80	5.05E-03
Biosynthetic process	2 527	72	40.18	+	1.79	3.71E-03
G-protein coupled receptor signaling pathway	1 539	6	24.47	–	0.25	3.27E-02
Sensory perception	1 281	3	20.37	–	<0.2	9.00E-03
Detection of stimulus	1 076	1	17.11	–	<0.2	2.80E-03

* *CC, cellular component; PC, protein class; BP, biological process*.

### CNVR genetic diversity analyses

Table 4 demonstrates pairwise population PhiPT values for CNVRs of seven cattle breeds. For all breed groups, the degree of variation within populations was considerably greater than that between populations. Pairwise population PhiPT values correspond to breed type groupings with Taurine breeds showing the least CNVR variation being captured. Sixteen and 84% of the CNVR genetic variation was among breeds and within breeds (Table 5).

**Table 4:** Summary results of AMOVA pairwise population CNVR PhiPT values for seven cattle breeds.

	**Afrikaner**	**Angus**	**Bonsmara**	**Drakensberger**	**Hereford**	**Nguni**	**Nguni Angus**
Afrikaner	0.000						
Angus	0.086	0.000					
Bonsmara	0.065	0.032	0.000				
Drakensberger	0.000	0.092	0.071	0.000			
Hereford	0.108	0.001	0.048	0.117	0.000		
Nguni	0.034	0.029	0.013	0.037	0.047	0.000	
Nguni Angus	0.601	0.341	0.524	0.000	0.400	0.558	0.000

**Table 5:** Summary AMOVA table demonstrating estimate among and within breed CNVR genetic variance for seven cattle breeds.

**Source**	**df**	**SS**	**MS**	**Est. Var**.	**(%)**
Among Pops	6	141.132	23.522	0.518	16
Within Pops	280	762.227	2.722	2.722	84
Total	286	903.359		3.241	100

**Figure 3:**
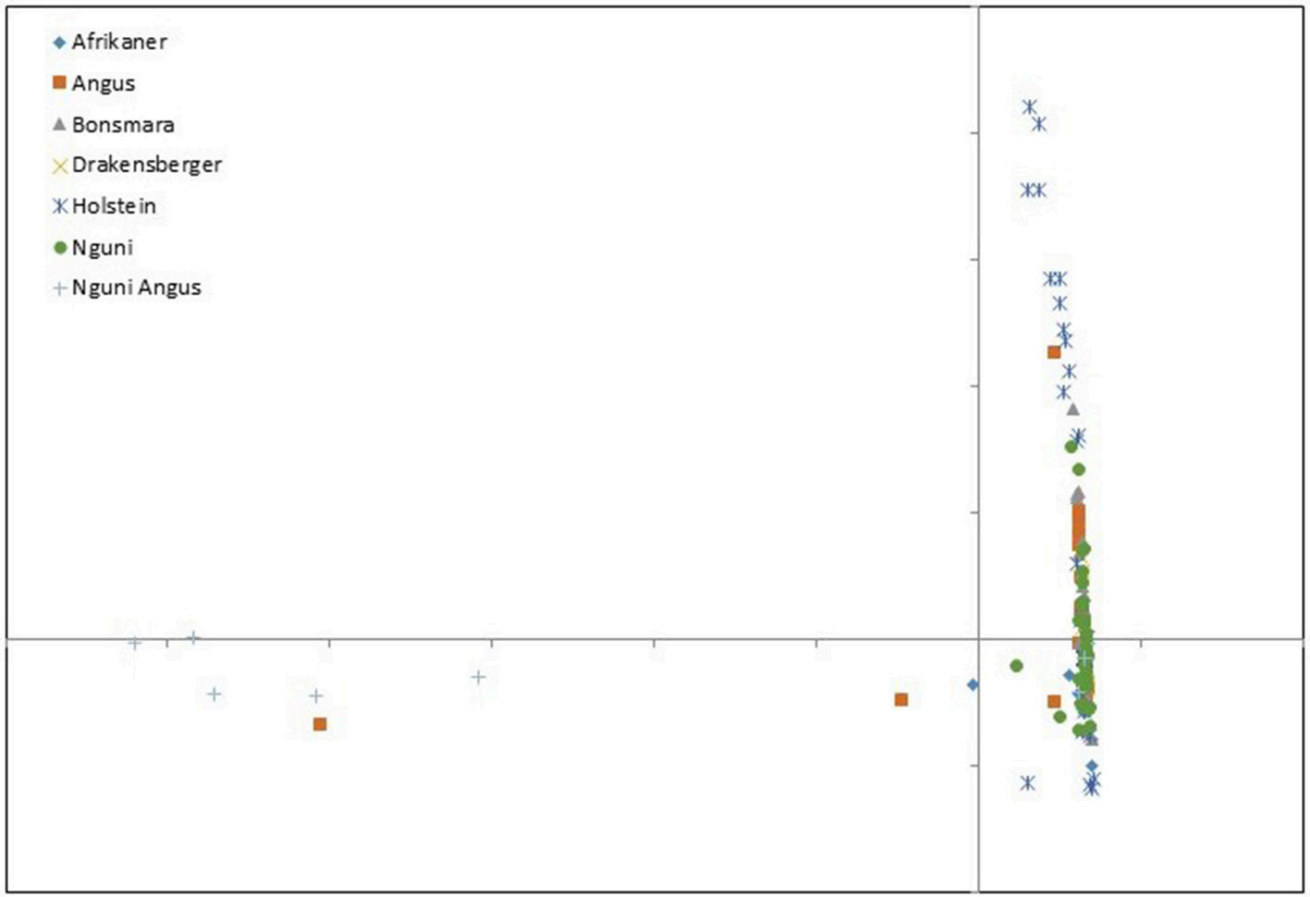
Principle components analyses for components 1 and 2 for CNVRs of animals from six different cattle breeds.

Principle component analysis demonstrated the greatest amount of variation to be captured in PC 1 with an eigenvalue of 221.267, explaining 87.45% of the total variation captured among individuals (Additional File 7). Principle component 11 demonstrated an eigenvector of 1.058 and was thus chosen as the cutoff component. The Nguni Angus cross animals were the most differentiated from the rest of the animals at PC1 against PC2 (Figure 3). With the exception of the Nguni Angus cross animals, all breeds clustered together. The Holstein animals clustered in the same region but with a larger spread. The Holstein animals pulled toward the top of the cluster, while the Angus and Afrikaner animals cluster more to the left. The Nguni, Drakensberger, and Bonsmara animals had the most compact clustering, pulling more to the right of the x-axis.

**Figure 4:**
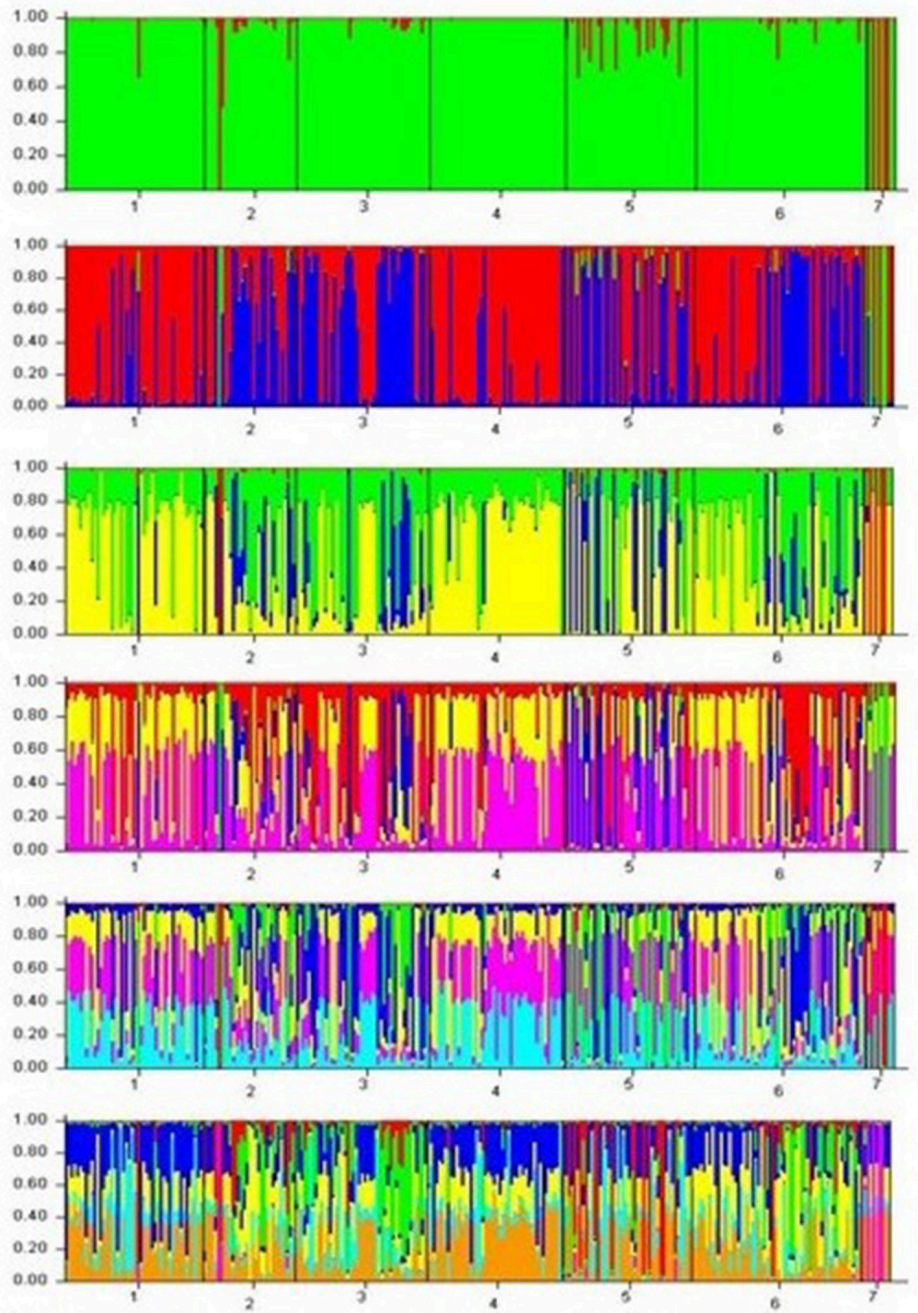
CNVR structure analyses for 287 animals from 7 different breeds of cattle for K = 3 to K = 8 (1 = Afrikaner; 2 = Angus; 3 = Bonsmara; 4 = Drakensberger; 5 = Holstein; 6 = Nguni; 7 = Nguni Angus).

STRUCTURE was utilized in R to depict the population structure of breed CNVRs presence. Figure 4 demonstrates the evolution of the population structure as K increased from 3 to 7. High levels of admixture were evident in the structure based clustering. At *K* = 3, genomic signatures distinct to the Nguni Angus crossbred animals were evident while genomic signatures distinct to the Sanga breeds of cattle (Afrikaner, Drakensberger and Nguni) were picked during progression to *K* = 8. Sanga cattle breeds comprise a crossbreed between indigenous Taurine and zebu cattle breed that are unique to Africa (Rege, 1999).

**Figure 5:**
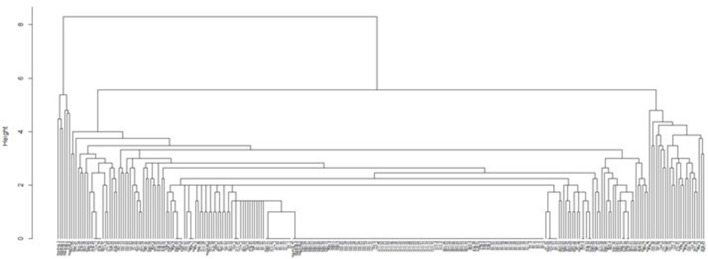
Hierarchal cluster analyses for CNVR presence of 287 cattle of seven cattle breeds.

A cluster dendrogram was generated from CNVRs identified in animals by means of R hclust (Figure 5). CNVRs for the most part clustered animals of the same breed together. Five of the 7 Nguni X Angus cross animals clustered together with 1 Angus animal in a clade distinct from the rest of the animals. A second clade was evident with a seemingly random mix of animals from different breeds with some animals clustering together within breeds, but others were seemingly random. The structure of the dendrogram suggest a disparity with some CNVRs being breed specific variations, while others may possibly be Bos taurus/Bos indicus CNVRs or possibly indicators of interindividual variation.

**Figure 6:**
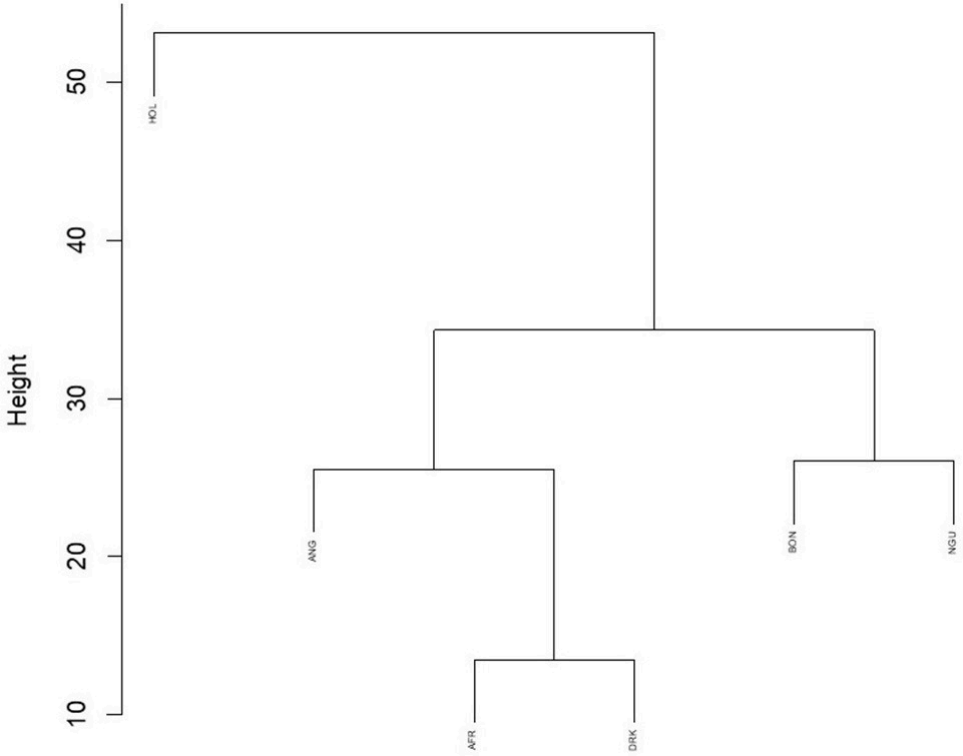
Hierarchal cluster analyses based on presence of CNVR in seven cattle breeds (HOL, Holstein; ANG, Angus; AFR, Afrikaner; DRK, Drakensberger; BON, Bonsmara; NGU, Nguni).

Hierarchal clustering analyses on CNVR frequency within breeds were performed. A cluster dendrogram of breeds is depicted in Figure 6. Binomial clustering of CNVR presence generated two distinct clades separating the indigenous pure breeds from the two Taurine breeds and the Nguni Angus crossbreed. CNVR presence within the Nguni Angus animals placed them right next to the Angus animals and completely separated from the Nguni. The two frequency plots, however generated distinctly different distributions. CNVR frequency articulated as a percentage caused the Holstein and Nguni Angus animals to segregate away from the other animals while the Angus breed moved to between the Bonsmara/Nguni and Afrikaner/Drakensberger clades. Upon using the number of animals presenting the CNVR the Nguni Angus breed was completely isolated while the two Taurine breeds clustered together and the indigenous breeds assembled in a stepwise fashion.

### CNVR gene ontology

Eight hundred and nine genes were covered by the 356 CNVRs identified across seven cattle breeds (Table 2). Drakensberger cattle had the least CNVR genes, while Angus had the most of the purebreeds and Nguni Angus had the most overall. Of the 809 genes, 6 genes [*low affinity sodium-glucose cotransporter-like* (*LOC527441*)*, netrin G2* (*NTNG2*)*, otopetrin 1* (*OTOP1*)*, solute carrier family 5 member 1* (*SLC5A1*)*, transmembrane protein 128* (*TMEM128*) and *WD repeat domain 1* (*WDR1*)] were common to all breeds. Three hundred and eighty nine CNVR genes were breed specific (Additional File 8). The most CNVR genes were shared between Angus and Nguni Angus animals. Afrikaner, Angus, Bonsmara, Drakensberger, Holstein, Nguni and Nguni Angus breeds had 17, 57, 26, 13, 19, 26, and 231 breed specific CNVRs. Heat shock proteins *HSPBP1* (*heat shock binding protein 1*)*, HSPB1* (*heat shock protein family B member 1*)*, HSPA5* (*heat shock protein family A* (*Hsp70*) *member 5*), and *HSP90AA1* (*heat shock protein 90 alpha family class A member 1*) considered to play a vital role in balancing immunity and survival during times of stress (Zhang Q. et al., 2014), were covered by CNVRs in Nguni, Angus, Holstein and/or Nguni Angus breeds. Severe reductions in WDR1 (WD40 repeat protein 1), identified in 42 animals from breeds in this study were reported to disturb megakaryocyte maturation and platelet shedding, aggravate neutrophilic autoinflammatory disease and trigger embryonic lethality in mice (Castellani et al., 2014). *LSP1* (*Lymphocyte-specific protein 1*) and *IGF-II* (*insulin-like growth factor 2*), covered by CNVRs identified in Angus and Nguni Angus animals and *IGLL1* (*immunoglobulin lambdalike polypeptide 1*) overlapped by CNVRs in 44 animals from all breeds except Drakensberger were differentially expressed in cattle selected for resistance or susceptibility to intestinal nematodes (Araujo et al., 2009). Other genes involved in immune response included *GSTT3* (*glutathione s-transferase theta-3*)*, GSTT1* (*glutathione s-transferase theta-1*), and *SMARCB1* (*SWI/SNF-related matrix-associated actin-dependent regulator of chromatin subfamily B member 1*) that were present in 35, 33, and 40 animals respectively from all breeds except the Drakensberger.

A *PANTHER* overrepresentation test using a Bonferroni correction for multiple testing was performed for genes covered by CNVR identified. Five GO biological processes, one molecular function and 25 cellular components demonstrated a significant (*p* < 0.05) over representation by CNVR genes and are presented in Additional File 9. Only Nguni, Holstein, Angus, and Nguni Angus breeds demonstrated breed specific over representation of 1, 15, 11, and 35 gene ontology processes, functions and/or components by CNVR genes respectively. Intracellular (GO:0005622), membrane-bounded organelle (GO:0043227), intracellular membrane-bounded organelle (GO:0043231), cytoplasm (GO:0005737), cytoplasmic part (GO:0044444), intracellular part (GO:0044424) where over represented by CNVR genes identified in Angus, Holstein, and Nguni Angus breeds.

## Discussion

CNVs are considered to play a role in breed formation and adaptation, with copy number differences occuring between breeds (Liu et al., 2010). Increasing evidence also suggests CNVs to play a primary role in interindividual diversity (Stankiewicz and Lupski, 2002; Sebat et al., 2004) attributed to both normal phenotypic variation and major variations in complex traits (Fellermann et al., 2006; Feuk et al., 2006). Great variation in the size and number of CNVRs has been reported in cattle (Hou et al., 2012; Jiang et al., 2012). In this study 1055 CNVs formed 356 CNVRs in 287 animals from 7 different cattle breeds representing Taurine, Sanga, Composite and cross bred breed groups using the Bovine 50 K Beadchip. Jiang et al. (2012) identified 367 CNVRs by means of PennCNV analyses of high-density SNP genotyping data from 96 Chinese Holsteins. Hou et al. (Hou et al., 2011) on the other hand, reports 682 CNVRs identified in 521 animals representing 21 different breeds also identified using Bovine50K SNP genotyping array. Discrepancies in CNVs and subsequent CNVRs between different breeds and even individuals could thus be expected. Although Jiang et al. (2013) highlight the differences in size and structure of populations, could also contribute to such incongruities. Hou et al. (2012) speculated that the distinctions in selected breeds for specific traits could be linked to specific CNVs. CNVR breed characterization, correlation analyses, population structure analyses and genetic diversity analyses all demonstrate the Taurine breeds and Sanga/Composite breeds to cluster in distinct groups with the Nguni Angus cross segregating completely alone. The two Taurine breeds presented noticeably more CNVRs than the indigenous and Composite breeds, coupled with a number of gene ontologies demonstrating overrepresentation. The greater number of CNVRs evident in the exotic Taurine breeds reflects findings of Choi et al. (2013) who compared the genome of a Hanwoo bull to that of Holstein and Black Angus respectively using whole genome sequencing methodologies. Narang et al. (2014) proposed that the migration and adaptation of a population or breed to a completely different environment to which they have typically been accustomed to, may require considerable changes on a genomic level that may be achieved via events like CNVs which may hence contribute toward adaptation. The introduction of exotic Taurine breeds to a new environment may have placed specific pressures on the genome, resulting in the formation of CNVRs at specific loci involved in processes, functions or components vital for adaptation. The greater number of CNVs present in the Taurine breeds, may suggest CNVs representing a response of the genome to selection pressures imposed by adverse climatic conditions on animals that have been bred for production and not necessarily for their innate ability to survive harsh conditions. Frequently encoding protein products that play a prominent role in species adaptation (Duda and Palumbi, 1999), segmental duplications are an important cause of genomic instability that results in nonallelic homologous recombination (NAHR) during meiosis and genomic innovations and are currently recognized as one of the major catalysts and hotspots for CNV formation (She et al., 2008; Alkan et al., 2009; Nicholas et al., 2009; Liu and Bickhart, 2012). This would hence explain the discrepancies between this and that of Choi et al. (Jiang et al., 2013) with Matukumalli et al. (2009) and Hou et al. (2011) who report Taurine breeds to have fewer CNVs than Composite, Indicine and African breeds. The African and Composite breeds in the study of Hou et al. (2011) were represented by fewer animals (39 and 46 respectively) and demonstrated an average of 7.21 and 7.17 CNVs per animal. This is not much more than the 6.23 average of 366 Taurine animals, but noticeably less than the 11.41 average of the 70 Indicine animals. Choi et al. (2013) suggested CNVs to be affected by recent intensive artificial selection schemes aimed at improving economically important production traits.

Similar to the findings of Molin et al. (2014), the majority of the CNVs identified in the present study were shared between fewer breeds with the most CNVs (30) being shared between Angus and Nguni Angus cattle (Additional File 2). Greater distinction can be drawn from breeds being grouped according to breed type. While genetic diversity analyses demonstrated the majority of CNVR variation to exist within population, the between diversity was least between breeds of the same type. The present studied demonstrated CNVR population structure segregating animals by breed type with Nguni Angus cross animals separating at *K* = b3 and the Afrikaner, Drakensberger, and Nguni breeds ghettoizing at *K* = 8. The evolution of the CNV population structure with increasing K values depicts breed history patterns with CNVs segregating breeds groups. The Drakensberger is considered to be one of the earliest Composite breeds developed. Its segregation with the Sanga type breeds is hence not surprising considering the possible role of adaptation on CNV prevalence. Although it was developed with a Taurine component, CNV evolution may reflect the selection pressures of adaptation that is evident in the Sanga breeds. Cicconardi et al. (2013), reported little variation in CNV distribution on chromosomes across five Italian cattle breeds, proposing CNV region (CNVR) variation to be greater between individuals than between breeds. Molin et al. (2014) identified 15 breed specific CNVRs out of 72 CNVs identified in 351 dogs from 30 different. CNVRs identified in a single breed may pose interest for the investigation into breed specific traits (Molin et al., 2014). This however differs from Zhang L. et al. (2014) who report lineage specific CNVRs, proposing CNVs in the Chinese cattle populations to be partly consequent to selective breeding during domestication but also subsequent to hybridization and introgression. Inadequately distinguishing between CNVRs that are breed specific and those that are bovine specific may be the cause of the significantly higher degree of variation being evident within populations (Table 5). We postulate that a large proportion of CNVRs are animal specific events, while only a few explicit CNVRs events to be exclusive to breeds. In addition to this, Figure 2 demonstrates breed specific CNVs sections within 4 large CNVRs that were detected in all 7 breeds. The delineation of CNVRs within this study may hence be responsible for low between breed diversity (Tables 4, 5) and high levels of CNVR admixture observed (Figure 4). Pienaar et al. (Pienaar, 2014) found high levels of within breed diversity for Afrikaner cattle using microsatellite data. Makina et al. (2014) found the Afrikaner breed to have the greatest number of alleles per locus when compared to the 5 other purebreeds in this study, while the Nguni had the least. Drakensberger cattle have the greatest genetic diversity of the 4 indigenous Sanga and Composite breeds, while the two Taurine breeds were reported to have had the greatest gene diversity (Makina et al., 2014). The Holstein and Angus breeds of the taurus cattle group have a longer history of artificial selection that has led to enhanced production (Choi et al., 2013). The observed discrepancies evident between some breeds could very well be caused by genetic drift due to bottlenecks, natural selection, and selective breeding (Hou et al., 2011). Itsara et al. (2010) determined different mutation processes to contribute disproportionately to CNVs dependant on the size of the *de novo* event. The mutation rate of CNVs has been established to be considerably higher than that of SNPs, with great variation in mutation rates occuring between loci (Campbell et al., 2011). The exact mutation and inheritance patterns of CNVs are, however not fully understood (Zhang L. et al., 2014). It has been proposed that forces such as recombination, selection, and mutations are the primry factors driving the genomic architecture of large variations (Jimenez, 2014), with CNVs comprising a mechanism by which the genome responds to selection pressures subseqeunt to genomic instability induced by such pressures (Redon et al., 2006). CNVRs correlations and breed type distribution observed in this study, further augment this theory exhibiting an external pressure acting on regions within the genome involved in specific functions (Table 3). Distinctions in CNVRs correlations specific to breeds and breed subpopulations, augments the notion that selection pressures play an important role in CNV formation (Hou et al., 2011; Porto-Neto et al., 2014). Twenty-two of the 110 CNVR loci present in more than 1 animal were utilized for CNVR correlation analyses and genetic diversity assessments. These constituted 74 significant correlations in all 7 breeds. Within the two exotic Taurine breeds, 906 significant CNVR correlations were determined, while only six significant CNVR correlations were identified in the indigenous Sanga and Composite breeds. Most of the associations were between CNVR loci of the same type. Taurine breed CNVR associations exhibited 866 deletion associations, 38 duplication associations, and 2 deletion and duplication associations. Deletions interrupt genes while also causing a loss of biological function and are therefore currently seen as the most common CNV effecting phenotype (Liu and Bickhart, 2012). Increased copy number may have a positive (McCarroll, 2008) or negative (Lee and Lupski, 2006) association with gene expression levels.

Composite breeds were developed from multiple breeds with the aim to combine the adaptive ability of the local breeds with the productive capabilities of the exotic breeds (Bonsma, 1980). The inclusion of the Composite breeds as well as the Taurine Sanga crossbreed in this study provided insight into the age and evolution of CNVs and the translation of CNVs when breed groups are amalgamated in a Composite breeds and cross breeds. The study of CNVs in crossbred and Composite breeds may hold clues in gaining greater insight into CNV formation and the possible role of CNVs in factors like hybrid vigor. The crossbred Nguni Angus animals, despite fewer animals, demonstrated considerably more CNVs than other breeds with distinct genomic signatures. This study comprises the first characterization of crossbred bovine animals. The noticeably higher number of CNVRs in these animals could indicate CNVRs to play a role in hybrid vigor. The Nguni Angus presents a popular cross in South Africa taking advantage of the strong maternal and adaptive characterstics of the Nguni and the production potential of the Angus.

CNVs may alter gene structure, dosage or gene functioning by disrupting coding sequences, long range regulation or by exposing recessive alleles (Zhang et al., 2009; Stankiewicz and Lupski, 2010; Liu and Bickhart, 2012). The phenotypic impact of CNVs is, however too a large extent related to the locations of the variant in relation to the genes (Buchanan and Scherer, 2008). Drakensberger cattle had the least CNVR genes, while Angus had the most of the purebreeds and Nguni Angus had the most overall. Only six genes were identified in all 7 South African breeds. The identification and breed distinctions of genes involved in processes vital for adaptation suggest CNVs to play a role in breed formation. Gene copy number is conventionally positively correlated with gene expression (Stranger et al., 2007), although cases of negative correlations have been reported (Lee and Lupski, 2006). A duplicated CNVR on chromosome 11 covering *AIF1L* (*allograft inflammatory factor 1-like*) and *ABL1* (*protein kinase abl1*) genes was correlated with a second duplication on chromosome 18 covering the *NLRP5* (*nacht, lrr and pyd domains-containing protein 5*) gene. The *AIF1L* is an important component of innate immunity and response to stress while *NLRP5* comprises part of the cellular defense response. *ABL1* gene mutations causes resistance to tyrosine kinase inhibitors which have been found to improve the management of chronic myeloid leukemia in humans (Shah et al., 2002; O'Hare et al., 2007). Of the six correlations present among CNVRs of the indigenous breeds, all except two were between duplicated regions. The only exceptions were correlations between a deletion on chromosome 6 and duplication on chromosome 29 and 26 respectively. Although no genes were covered by the deleted CNVR, the correlated duplication on chromosome 29 covered 24 genes including *TSPAN32* (*tetraspanin-32*)*, CDKN1C* (*cyclin-dependent kinase inhibitor 1*) and *TNNT3* (*troponin T, fast skeletal muscle*) involved in a variety of biological processes, molecular functions and cellular components.

## Conclusion

Three hundred and fifty-six Unique CNVRs were identified in 287 animals from 2 Taurine, 2 Composite, 2 Sanga, and 1 Sanga Taurine cross Cattle breeds using the Bovine 50 K Beadchip. A number of cellular components, molecular functions and biological processes demonstrated overrepresentation by genes covered or lying within 10 Mb of CNVRs identified. Correlations between CNVR presence was evident, with considerably more CNVR correlations occurring among the commercially bred Taurine breeds. Such correlations suggest selection pressures being exerted on different genomic regions involved in specific processes and functions. CNVs may be a means by which the genomes respond to selection pressures and subsequently adapts. Variations in CNVR presence between breeds was present with more CNVRs being present in the Nguni Angus cross and the two Taurine breeds. Composite and cross bred animals demonstrated the most within breed CNVR variation, while Sanga cattle demonstrated the least. The Nguni Angus cross demonstrated unique CNV genetic signatures, while some CNVs segregated in both the Taurine and Sanga breeds to some degree. This study indicatesd CNVRs to play a role in both interindividual and between breed variations. With Sanga and Taurine breeds having undergone different selection pressures, the variation in CNV incidence between these groups combined with the CNV correlations designate CNVRs to be genomic features prevalent in selection and adaptation. The distinct properties of CNVRs in the Nguni Angus cross animals need also be explored with possible implications in events like hybrid vigor.

## Data availability statement

Datasets supporting the conclusions of this study will be made available by the authors, without undue reservation, to any qualified researcher.

## Ethics statement

Genomic data was obtained from Makina et al. (2014, 2015). The Agriculture Research Council, who generated the data published by Makina et al. (2014, 2015), granted permission to use the data in the present analyses.

## Author contributions

Molecular genetic, bioinformatics, and statistical analyses were performed by MP who also drafted the manuscript. FM concieved of the study, aided the analyses of the data, and participated in the design and structure of the manuscript. KD participated in the coordination and preparation of the manuscript. All authors read and approved the final manuscript.

### Conflict of interest statement

The authors declare that the research was conducted in the absence of any commercial or financial relationships that could be construed as a potential conflict of interest.

## Supplementary material

Additional File 1 Table depicting the count, minimum (MinL), maximum (MaxL) and average (AvL) lengths and total length (LN) of CNVRs identified on each of the 29 chromosomes of 287 cattle from seven different breeds.

Additional File 2 The number of CNVs (Num CNVs) and the CNVs identified that were present in more than one of the seven cattle breeds (BRDs).

Additional File 3 Significant pairwise association Chi^2^ and *P*-values of deletion and duplication (CN_A and CN_B) CNVR events (CNVR_LocA and CNVR_LocB) identified in all seven cattle breeds.

Additional File 4 Associated CNVRs and number of animals (IND) in which they were identified across seven cattle breeds.

Additional File 5 Significant pairwise association Chi^2^ and P-values of deletion and duplication (CN_A and CN_B) CNVR events (CNVR_LocA and CNVR_LocB) identified in 2 South African Taurine cattle breeds.

Additional File 6 Significant pairwise association Chi^2^ and P-values of deletion and duplication (CN_LocA and CN_LocB) CNVR events (CNVR_LocA and CNVR_LocB) identified in indigenous South AfricanSanga and Composite cattle breeds.

Additional File 7 Eigen values (EIV) of *first* 11 Principle Components (PC) generated from a genetic distance matrix of 277 animals (AN) from seven cattle breeds (BRD).

Additional File 8 Table demonstrating the overlap (Num GEN) of CNVR genes (GEN) identified in seven cattle breeds (BRD).

Additional File 9 Complete GO molecular functions (MF), cellular components (CC), and biological processes (BB) with significant (*p* < 0.05) over enrichment by genes covered by CNVRs in seven cattle breeds.
