# Corrigendum: Genetic Diversity of Seven Cattle Breeds Inferred Using Copy Number Variations

**DOI:** 10.3389/fgene.2018.00252

**Published:** 2018-07-13

**Authors:** Magretha D. Pierce, Kennedy Dzama, Farai C. Muchadeyi

**Affiliations:** ^1^Animal Production, Agricultural Research Council, Pretoria, South Africa; ^2^Department of Animal Sciences, University of Stellenbosch, Stellenbosch, South Africa; ^3^Biotechnology Platform, Agricultural Research Council, Pretoria, South Africa

**Keywords:** genetic diversity, CNVs, population structure, South African cattle, breed history, selection

In the original article Makina et al. (2015) was not cited in the article. The citation has now been inserted in Materials and Methods, Sample Collection and Genotyping, paragraph 1 and should read:

Genomic data was obtained from Makina et al. (2014) and Makina et al. (2015). This comprised 287 animals comprising of two Taurine (45 Holstein and 32 Angus), two Sanga (59 Nguni and 48 Afrikaner), two Composite (46 Bonsmara and 48 Drakensberger) and one crossbred (10 Nguni Angus) breeds sampled from throughout South Africa. Informed consent from respective breeders was obtained. The protocol utilized for the collection of samples, DNA extraction and genotyping has been published (Makina et al., 2014, 2015).

Similarly, the protocol utilized for the collection of samples, DNA extraction and genotyping has been published (Makina et al., 2014). Animal handling and sample collection were performed according to the University of Pretoria Animal Ethics Committee code of conduct (E087-12).

A correction has been made to Materials and Methods, Sample Collection and Genotyping, paragraph 1:

The protocol utilized for the collection of samples, DNA extraction and genotyping has been published (Makina et al., 2014, 2015).

Ethics approval was obtained for the study (Ref. Nr.: 2014/CAES/101).

Finally, we neglected to include information regarding ethical approval for this study (Ref. Nr.: 2014/CAES/101). A correction has been made to Ethics Statement, paragraph 1:

Genomic data was obtained from Makina et al. (2014, 2015). The Agriculture Research Council, who generated the data published by Makina et al. (2014, 2015), granted permission to use the data in the present analyses.

The authors apologize for these errors and state that this does not change the scientific conclusions of the article in any way.

The original article has been updated.

## Conflict of interest statement

The authors declare that the research was conducted in the absence of any commercial or financial relationships that could be construed as a potential conflict of interest.
